# Novel Reassortant H3N2 Avian Influenza Virus Isolated from Domestic Ducks in Eastern China in 2016

**DOI:** 10.1128/genomeA.01237-17

**Published:** 2017-11-30

**Authors:** Wenqiang Sun, Jiaxin Li, Jiao Hu, Daxiu Jiang, Zhichuang Ge, Chaonan Xing, Xiaoquan Wang, Min Gu, Xiaowen Liu, Shunlin Hu, Xiufan Liu

**Affiliations:** Animal Infectious Disease Laboratory, School of Veterinary Medicine, Yangzhou University, Yangzhou, Jiangsu, China; Jiangsu Co-Innovation Center for Prevention and Control of Important Animal Infectious Diseases and Zoonosis, Yangzhou University, Yangzhou, Jiangsu, China; and Key Laboratory of Prevention and Control of Biological Hazard Factors (Animal Origin) for Agri-Food Safety and Quality, Ministry of Agriculture of China, Yangzhou University, Yangzhou, Jiangsu, China

## Abstract

H3 subtype avian influenza virus (AIV) poses a great threat to public health, and so investigating its epidemiology is of great importance. A novel reassortant H3N2 AIV strain was isolated from a live poultry market in eastern China. The strain’s genes originated from H1N1, H3, and H7 AIVs. Thus, the genome information of the H3N2 isolate will help to investigate further the epidemiology of H3 subtype AIVs in China.

## GENOME ANNOUNCEMENT

Avian influenza viruses (AIVs) have a wide range of hosts, from birds to mammals (1–7). Notably, multiple subtypes of influenza viruses (H3, H4, H6, H9, H10, etc.) have also circulated and evolved in live poultry markets (LPMs), which are considered to be recombinant sources of AIVs in China (8–10). Meanwhile, highly pathogenic AIVs (HPAIVs) and low pathogenic AIVs (LPAIVs) frequently undergo reassortment with each other (7–9, 11–13). In particular, H3 subtype AIVs can be detected in LPMs in China (9). As we know, H3 subtype AIVs have a wide host range, including birds, humans, pigs, dogs, and horses (3, 4, 14–17). H3 subtype AIVs can serve their gene segments to other HPAIVs and also reassort with other AIV subtypes (10, 18–20). So, the monitoring of H3 subtype AIVs is of great significance to public health.

An H3N2 strain of AIV was isolated from a duck in September 2016 in Jiangsu, China, and named A/duck/Jiangshu/YZ916/2016(H3N2). RT-PCR was performed by universal primers (21), followed by sequencing (Nanjing Genscript). The DNA sequences were edited with Lasergene version 7.1 software (DNASTAR), and genetic evolution analysis of the isolate was performed with MEGA7 software.

The complete genome of the isolate included eight genes, polymerase basic 2 (PB2), polymerase basic 1 (PB1), polymerase acidic (PA), hemagglutinin (HA), nucleoprotein (NP), neuraminidase (NA), matrix (M), and nonstructural (NS) proteins. The open reading frame lengths of these segments are 2,280, 2,277, 2,151, 1,701, 1,497, 1,401, 982, and 838 nucleotides, respectively. PEKQTR↓G at the cleavage site of the isolate indicated that the virus was an LPAIV. The potential glycosylation sites of the isolate revealed that there were five potential N-linked glycosylation sites in HA (positions 22, 38, 165, 285, and 483) and six in NA (positions 61, 69, 86, 146, 200, and 402). Moreover, there were amino acid residues, Q226 and G228, at the receptor binding site in the HA protein, which indicated that the isolate preferentially binds to a receptor of avian origin. In PB2, there were no mutations for E627K and D701N, which are associated with mammalian host adaptation (22).

Genetic evolution analysis of the eight genes of the isolate indicated that the HA gene showed a high similarity (97%) with isolate A/duck/Jiangsu/J2186/2014(H3). The NA gene showed high homology (99%) with isolate A/chicken/Ganzhou/G243/2016(H3N2). The PB2 gene showed the highest homology (98%) with isolate A/duck/Jiangxi/21224/2013(H7N3). The PB1 gene showed the highest homology (99%) with isolate A/chicken/Huzhou/3916/2013(H7N3). The PA gene showed the highest homology (99%) with isolate A/duck/Mongolia/520/2015(H1N1). The NP gene showed the highest homology (99%) with isolate A/duck/Jiangxi/21710/2013(H7N3). The M gene showed the highest homology (99%) with isolate A/muscovy duck/Vietnam/LBM348/2013(H3N8). The NS gene was most closely related to isolate A/environment/Korea/W478/2014(H7N7) with 99% homology. Overall, the isolate was a novel reassortant H3N2 AIV originating from H1N1, H3, and H7.

H3 subtype AIVs can pose a great threat to public health, and thus the genome information reported here can help further epidemiological investigations of H3 subtype AIVs in China.

### Accession number(s).

This genome sequence has been deposited in GenBank under the accession numbers MG021165 to MG021172.
